# Is IIIG9 a New Protein with Exclusive Ciliary Function? Analysis of Its Potential Role in Cancer and Other Pathologies

**DOI:** 10.3390/cells11203327

**Published:** 2022-10-21

**Authors:** María José Oviedo, Eder Ramírez, Manuel Cifuentes, Carlos Farkas, Andy Mella, Romina Bertinat, Roberto Gajardo, Luciano Ferrada, Nery Jara, Isabelle De Lima, Fernando Martínez, Francisco Nualart, Katterine Salazar

**Affiliations:** 1 Laboratory of Neurobiology and Stem Cells, NeuroCellT, Department of Cellular Biology, Faculty of Biological Sciences, University of Concepcion, Concepcion 4070386, Chile; 2 Department of Cell Biology, Genetics and Physiology, University of Malaga, IBIMA, 29071 Malaga, Spain; 3 Networking Research Center on Bioengineering, Biomaterials and Nanomedicine, (CIBER-BBN), 29071 Málaga, Spain; 4 Biomedical Sciences Research Laboratory, Department of Basic Sciences, Faculty of Medicine, Universidad Católica de la Santísima Concepción, Concepcion 4090541, Chile; 5 Institute of Natural Sciences, Faculty of Veterinary Medicine and Agronomy, Universidad de Las Américas, Santiago 7500975, Chile; 6 Integrative Center of Biology and Applied Chemistry (CIBQA), Universidad Bernardo O’Higgins, Santiago 8370854, Chile; 7 Departament of Pharmacology, Faculty of Biological Sciences, University of Concepcion, Concepcion 4070386, Chile; 8 Center for Advanced Microscopy CMA BIOBIO, Faculty of Biological Sciences, University of Concepcion, Concepcion 4070386, Chile

**Keywords:** IIIG9, protein phosphatase 1, adherens junctions, ependymal cells, hydrocephaly, ciliopathies, ependymoma

## Abstract

The identification of new proteins that regulate the function of one of the main cellular phosphatases, protein phosphatase 1 (PP1), is essential to find possible pharmacological targets to alter phosphatase function in various cellular processes, including the initiation and development of multiple diseases. IIIG9 is a regulatory subunit of PP1 initially identified in highly polarized ciliated cells. In addition to its ciliary location in ependymal cells, we recently showed that IIIG9 has extraciliary functions that regulate the integrity of adherens junctions. In this review, we perform a detailed analysis of the expression, localization, and function of IIIG9 in adult and developing normal brains. In addition, we provide a 3D model of IIIG9 protein structure for the first time, verifying that the classic structural and conformational characteristics of the PP1 regulatory subunits are maintained. Our review is especially focused on finding evidence linking IIIG9 dysfunction with the course of some pathologies, such as ciliopathies, drug dependence, diseases based on neurological development, and the development of specific high-malignancy and -frequency brain tumors in the pediatric population. Finally, we propose that IIIG9 is a relevant regulator of PP1 function in physiological and pathological processes in the CNS.

## 1. Introduction

IIIG9 is a poorly characterized protein. This protein is encoded by the *PPP1R32* gene, also known as *C11orf66, FLJ32771, 4930579J09Rik, AU015816,* and *MGC144717*. The human IIIG9 orphan gene (*C11orf66*) is located at the 11q12.2 locus, and its mRNA expression was first reported in 2002, when high expression was demonstrated in the ependymal wall of the fourth cerebral ventricle [1]. In rats, IIIG9 exists as two transcripts, IIIG9L (1491 bp) and IIIG9S (1355 bp), which leads to the emergence of 426 and 381 amino acid proteins, respectively. Both isoforms differ by 44 amino acids that are present in the *N*-terminal region of IIIG9L but absent in IIIG9S [1]. In humans, there are two IIIG9 isoforms: one of 425 amino acids (1532 bp) and the other of 405 amino acids (1472 bp) [2]. The short isoform does not have the sequence found between amino acids 230 to 249 (UniProt data), which are present in the long isoform. In mice, even when two transcripts are generated (1511 bp and 1515 bp), only a single protein of 427 amino acids is reported [3].

Previous studies have shown that the IIIG9 protein is mainly localized to the cilia of the trachea, fallopian tube epithelium, spermatozoon tail, and ependyma [4,5,6], suggesting a major involvement in regulating motile cilia under normal conditions. In this review, we summarize detailed information on IIIG9, its cellular expression, and its relationship with different human pathologies, with a focus on ependymoma tumors.

## 2. Materials and Methods

Model structure of human PPP1R32: The full model of Human PPP1R32 was obtained from the C-I-TASSER server [7,8] by submitting the PPP1R32 amino acid sequence deposited in UniProtKB (accession Q7Z5V6). We conducted molecular dynamics simulations on the modeled structure with GROMACS (version 2019.1) [9], employing an OPLS-AA/L all-atom force field [10]. We minimized atom clashes in the system using the steepest descent method until potential energy was below 1000 kJ/(mol×nm). We considered a cutoff of 1.0 nm for non-bonded interactions under periodic boundary conditions (PBCs). Then, we conducted an NVT ensemble (constant Number of particles, Volume, and Temperature) with no pressure coupling and a modified Berendsen thermostat at 300 K. We employed the Parrinello–Rahman barostat in the NPT ensembles to keep the constant pressure at 1 bar and the modified Berendsen thermostat to keep the temperature constant at 300 K. Production dynamics were analyzed for 700 nanoseconds using the leap-frog algorithm with an integration step of 2 femtoseconds as the motion setting. Models every 100 nanoseconds were evaluated using the ProSA webserver [11,12], and the best model was selected according to the Z-score (600 nanoseconds). The C-I-TASSER [7,8] and the resulting structures from the molecular dynamic simulations surpassed the quality of the current PPP1R32 structure provided by AlphaFold [13,14,15], as assessed by the ProSA webserver.

## 3. IIIG9 Is a Regulatory Subunit of Protein Phosphatase 1 (PP1)

**Figure 1:**
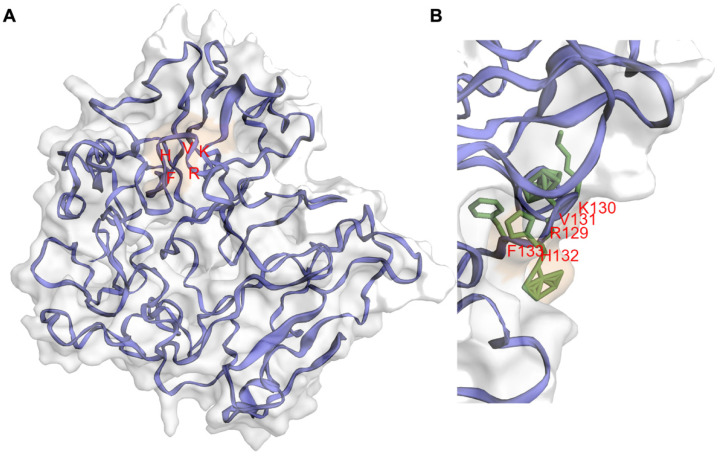
Molecular dynamics-refined model structure of human PPP1R32. (**A**) Cartoon representation of the best human PPP1R32 model according to the Z-score assessed by the ProSA webserver after 700 nanoseconds of molecular dynamics simulations. Protein structure, side chains, and surface patches were rendered with the EzMol server [18,19]. The model can reach a globular stable structure after 400 nanoseconds of molecular dynamics simulations. In the PPP1R32 protein model, the RKVHF motif (depicted with red letters) is accessible to the solvent. (**B**) Snapshot of RKVHF motif, depicting a histidine residue fully accessible to the solvent.

IIIG9 was initially described as a protein of unknown function because it showed no significant homology to any known protein [1]. Additionally, according to the GCG Program ‘Motifs’ algorithm, it was not possible to identify any hydrophobic amino acid clusters indicative of signal sequences or transmembrane regions [1]. However, the rat IIIG9 protein sequence contains significant matches with sequences in other species such as the chicken and zebrafish [1]. Recently, with the aid of residue contact maps and artificial intelligence, it has become possible to generate a three-dimensional structure model of IIIG9 through the use of the C-I-ITASSER server [7,8]. After over 600 nanoseconds of molecular dynamics, IIIG9 reached a stable globular structure with the absence of large secondary structures and the presence of short alpha helices, in which the RKVHF sequence can be located. There are also a few beta-sheets at the level of the *N*-terminal region and a destructured region in the *C*-terminal region (Figure 1). The lack of secondary structures as intrinsically disordered proteins and the presence of the RKVHF sequence characterize more than 70% of PP1 regulatory proteins, which allow them to adopt highly flexible, transient conformations, thus promoting interaction with the protein surface of PP1 (AlphaFold: Protein Structure Database) [13,14,15]. The RKVHF sequence is a short, degenerate domain present between amino acids 129 and 133 (long isoform) or 85 and 89 (short isoform) in rat proteins or between amino acids 129 and 133 in both human and mouse isoforms [16] (Figure 1). Additionally, the interaction of IIIG9 with PP1 has been validated in vitro and then used in in silico screening reports for proteins that present an RVxF-type binding domain [16,17]. In conclusion, IIIG9 was identified as a candidate protein that interacts with PP1.

## 4. Regulation of PP1 by IIIG9

PP1 is a ubiquitous and highly conserved phosphatase, targeting about half of the proteins of eukaryotic cells. It is thought to cleave the ester connection of phosphates linked to serines or threonines [20]. In mammals, there are multiple PP1 catalytic subunits (α, β, and γ), and their locations and functions differ based on their interactions with more than 200 regulatory protein subunits [17,21]. In humans, three isoforms of PP1α have been described, including a 330 aa (1421 bp) canonical sequence and two isoforms produced by alternative splicing (both computationally predicted) of 286 (1289 bp) and 341 amino acids (1454 bp) (UniProt). The PP1β catalytic subunit has two transcripts of 4916 bp and 4925 bp, which lead to a single isoform of 327 amino acids. Finally, two alternative splicing isoforms have been described for PP1γ: PP1 gamma 1 (2552 bp) and PP1 gamma 2 (1521 bp), with 323 and 327 amino acids, respectively. As with all highly dynamic and specific regulatory proteins, PP1 plays an important role in cellular processes (such as the cell cycle, protein synthesis, and transcription), as well as pathologies (such as cancer, heart disease, memory loss, type 2 diabetes, and viral infections) in which it has potential therapeutic roles [22,23,24].

PIPs (PP1-interacting proteins) interact with the different PP1 regulatory subunits through short sequences, which create PP1 holoenzymes with unique properties, a strategy that has been defined as a molecular “lego” that governs specificity [17]. Initially, the RXvF-like motif was defined as a region of five amino acids with the consensus sequence of [K/R]-X_(0,1)_-[VI]-(P)-[FW], where X is any residue and P is any residue except proline [25]. However, the sequence is summarized as [KR]-[KR]-[VI]-(FIMYDP)-[FW], where the most conserved residues are 1, 3, and 5; at position 4, the following amino acids are never found: Phe (F), Ile (I), Met (M), Tyr (Y), Asp (D), and Pro (P) [16]. However, this sequence is mostly enriched for R (17%), K (11%), S (21%), and T (18%). The presence of S and T is decisive in the interaction between PP1 and PIPs because the phosphorylation of these residues disrupts the union with PP1 [16,26]. The docking of the RVxF domain does not affect the catalytic activity of PP1, but it does increase the concentration of the interactor, which alters the substrate specificity of PP1. On the other hand, crystallographic studies have shown that the RVxF domain of a PIP is found in a flexible loop that adopts an extended beta-sheet by binding to the hydrophobic pocket of PP1, which is 20 Å farther from the catalytic site [16,26]. According to the structure predicted in AlphaFold, IIIG9 exhibits the RKVHF sequence between amino acids 129 to 133 at the level of a short helix present in the human sequence. Furthermore, this sequence lacks the S/T amino acids at position 4, indicating that the interaction between IIIG9 (Figure 1) and the catalytic subunits of PP1 is not regulated by phosphorylation/dephosphorylation. Although some PP1 regulatory proteins specifically interact with a single PP1 isoform, others bind all the catalytic subunits of this phosphatase, at least in vitro [17]. Double hybrid assays performed with a cDNA library derived from human testes and using a bait for the union of the PP1γ1 and PP1γ2 isoforms (generated by tissue-specific alternative splicing of the PP1γ gene) demonstrated that human IIIG9 interacts with both isoforms [27]. In turn, the colocalization of IIIG9 and PP1γ has been observed in spermatogonia and mature spermatozoa from mice and bovines, respectively [28]. In addition, the interaction between IIIG9 and the alpha subunit of PP1 (PP1α) has been demonstrated via co-immunoprecipitation studies on HEK293 cell extracts that overexpress C11orf66-GFP (human IIIG9 fused to GFP) and the use of yeast co-transformation assays [16,27].

The identification of new regulatory subunits of the catalytic subunits of PP1 will allow us to understand how the functional diversity of this phosphatase is regulated in eukaryotic cells. In addition, the presence of numerous PIPs in concentrations that are in large molar excess prevents PP1 from being free, causing uncontrolled dephosphorylation that leads to cell death [29]. Additionally, the characterization of the expression and function of new PIPs, such as IIIG9, will make it possible to define the molecular interactome of PP1 around a certain cellular process, such as during the formation of cell polarity where PP1 participates in signaling mechanisms with Cdc42/Par-3/aPKC/Par6 [16], opening new therapeutic targets that specifically regulate the action of PP1. Other interaction motifs located near the *N*-terminus of the RKVHF sequence, such as MyPhoNE, RxxQ[VIL][KR]x[YW], SILK [GS]IL[RK], SpiDoC [17], and IDoHA [17], have been characterized in other PIPs, but these are not present in IIIG9 [16].

## 5. Other Possible IIIG9-Interacting Proteins

**Figure 2:**
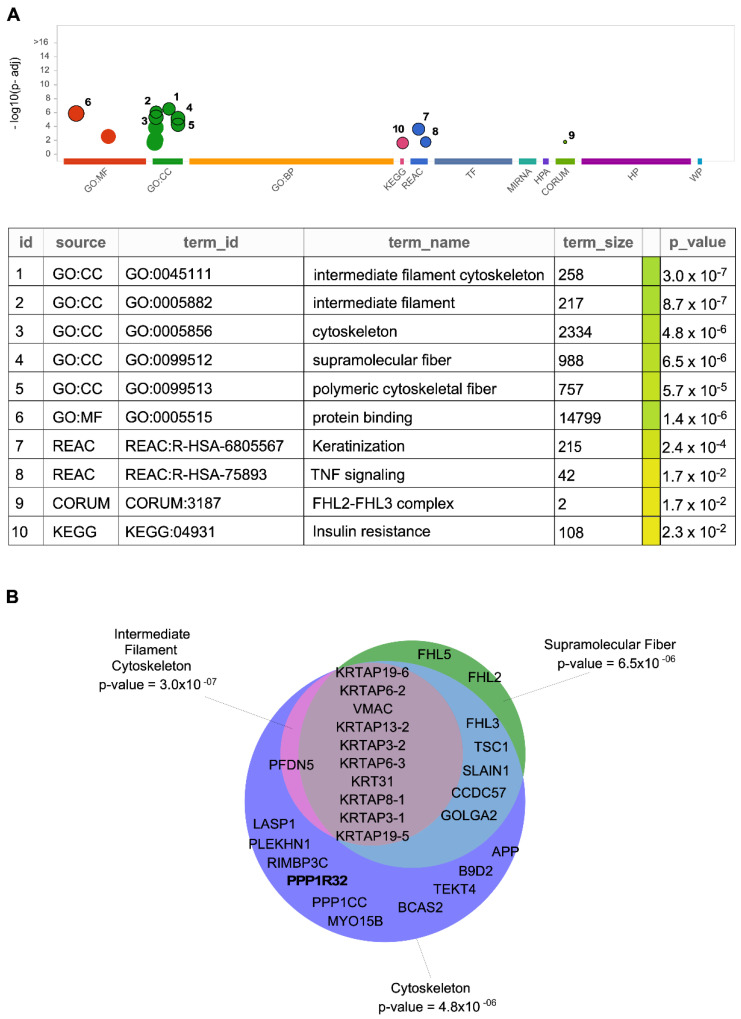
IIIG9 interactors obtained from BioGRID grouped by biological process. (**A**) Manhattan plot of g: Profiler for IIIG9 interactors: the x-axis shows functional groups by data source (Gene Ontology (GO): Molecular Function, Cellular component, Biological Process; Kyoto Encyclopedia of Genes and Genomes (KEGG); Reactome (REAC); Protein complex (CORUM)) and the y-axis shows the significance in each group. The *p*-values in the table indicate low (yellow) and high (blue) significance. (**B**) Venn diagram indicates the subcellular localization of IIIG9 interactors, with respective *p*-values [42].

Information regarding the interaction of IIIG9 with other proteins and its role in physiological and pathological processes is still limited. However, analyzing the binary interactions of 17,500 proteins enabled the generation of the first human reference interactome (HuRi) map [30,31]. These open-access datasets allowed us to identify putative interactors for IIIG9 [32,33], which were summarized according to the Database of Protein, Genetic and Chemical Interactions, BioGRID (Table 1 and Figure 2) [34,35].

**Figure 3:**
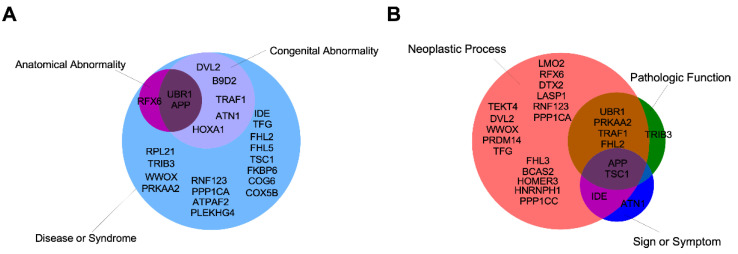
IIIG9-interactor proteins organized into semantic groups (UMLS^®^) of diseases extracted using DisGeNet (**A**,**B**) Interactors were experimentally validated (yeast via two-hybrid and affinity capture—MS methods). The semantic groups correspond to concept unique identifiers from the Unified Medical Language system^®^ (UMLS) Metathesaurus ^®^ (version UMLS 2018AA).

Importantly, these proteins could be grouped by their known biological functions, allowing us to infer new cellular roles for IIIG9 in physiological and pathological contexts (Figure 3 and Supplementary Table S1) [30,43,44,45,46,47,48].

One identified IIIG9 interactor is transcriptional regulatory factor 6 (RFX6), a transcription factor that participates in the differentiation of pancreatic beta cells and insulin secretion. Mutations in this gene are associated with Mitchell–Riley syndrome, characterized by neonatal diabetes with pancreatic hypoplasia, duodenal/jejunal atresia, and gallbladder agenesis [49]. The first findings describing the function of RFX were in *C. elegans*, where daf-19 (the only gene that encodes RFX in this organism) is a crucial regulator of ciliogenesis that controls the transcription of proteins that participate in cilium assembly via intraflagellar transport [50,51]. In vertebrates, RFX family members participate in assembly and cilia motility [52], and their dysfunction impairs cilia development; RFX3 has been implicated in developmental disorders such as ciliopathies [53].

Another possible interaction of IIIG9 with atrophin-1 protein (ATN1) was also observed. ATN1 is related to dentatorubral-pallidoluysian atrophy (DRPLA), a rare neurodegenerative disorder characterized by cerebellar ataxia, myoclonic epilepsy, choreoathetosis, and dementia [44,54,55].

Additionally, the PDZ domain is highly relevant in the maintenance of protein–protein interactions. The *C*-terminal region of IIIG9 is capable of binding to the PDZ domains of the E3 ubiquitin ligase, LNX1 [56], through a non-canonical PDZ-binding domain that contains a short sequence of amino acids. The data suggest that the IIIG9 concentration could be regulated by the ubiquitination by LNX1 and other ubiquitin ligases. However, this hypothesis needs to be experimentally proven.

Thus, the interaction of IIIG9 with these proteins and others that are part of the interactome could potentially reveal novel regulatory roles for phosphatases in the aforementioned pathologies, eventually resulting in future therapeutic targets.

## 6. Expression of IIIG9 in Human Cell Lines and Adult Tissues

The expression of IIIG9 in human cell lines can be reviewed in “The Human Protein Atlas”. IIIG9 is mainly detected in ciliated epithelial cells from the bronchi, the fallopian tube, and the nasopharynx, as well as early and late spermatids of the seminiferous tubules of the testes [4,57]. In addition, the expression of IIIG9 mRNA has been observed in different cell lines of neuroepithelial stem cells derived from human iPSCs (AF22), from pancreatic cancer (CAPAN-2), immortalized from the retina (hTERT-RPE1), immortalized from the mammary gland (hTERT-HME1), from certain skeletal muscles (HSkMC), from adipose tissue (ASC TERT1), and in some tumor cell lines derived from lymphoid organs (JURKAT, Karpas-707, and U266/70) and myeloids (THP-1 and U-937).

**Figure 4:**
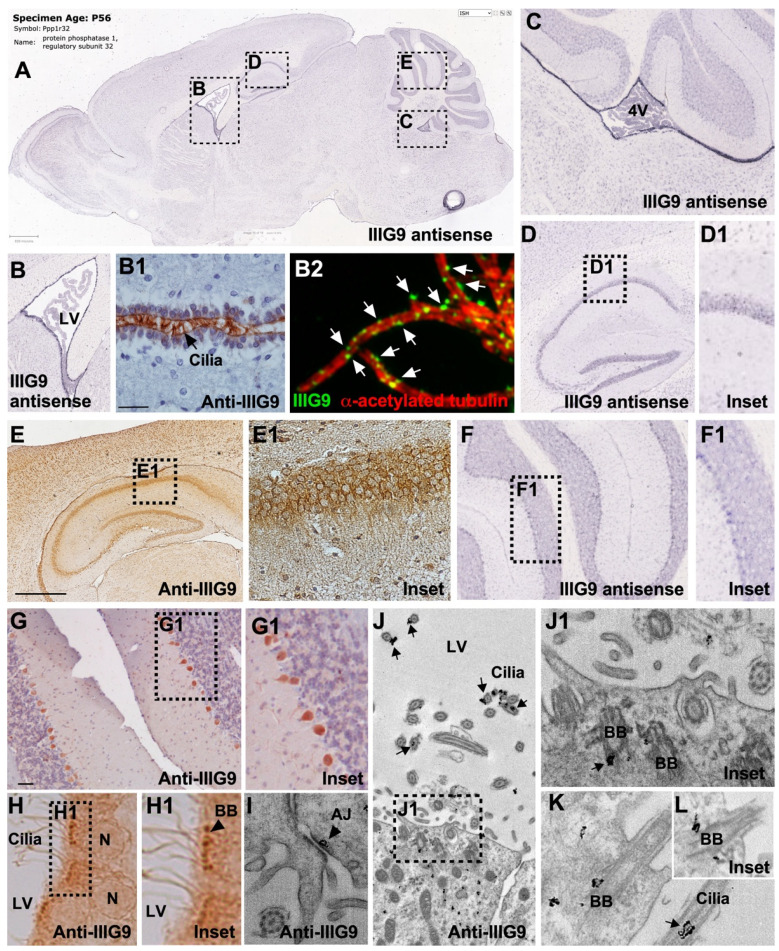
Comparison between IIIG9 mRNA expression and protein localization in the adult brain. (**A**) In situ hybridization analysis in adult mouse brain reported in Allen Brain Atlas for the *PPP1R32* gene. (**B**,**C**,**D**–**D1**,**F**–**F1**) High-magnification image for antisense riboprobe in ependymal cell from lateral and fourth ventricles, neurons from hippocampus, and Purkinje cells from the cerebellum. (**B1**,**E**–**E1**,**G**–**G1**) Immunohistochemical characterization of IIIG9 showing a positive reaction in the cilia and apical membrane of ependymal cells from the dorsal third ventricle and in the soma of hippocampal and Purkinje neurons. (**B2**). Super-resolution SIM showing IIIG9 dotted pattern (green, arrows) and alpha acetylated tubulin cilia (red) from isolated rat ependymal cells. Sagittal and frontal sections of mouse brain were immunostained with anti-IIIG9 (1:500) and a secondary antibody conjugated with peroxidase. (**H**–**L**) Immunohistochemical analysis using anti-IIIG9 antibody and anti-IgG labeled with 10 nm gold particles. (**H**–**H1**) In semi-thin sections, IIIG9 is detected in basal bodies of ependymal cilia (arrow). (**I**–**L**) In ultra-thin sections, IIIG9 was detected in adherens junctions (arrow), between the ring of nine outer microtubule duplets and the ciliary membrane (arrows), and in the basal bodies (arrow). Magnification in (**H**): ×100. Scale bars: **A**: 839 µm; (**B1**): 50 µm; (**E**): 1 mm; (**G**): 60 µm; (**J**): 1 µm. (**B**–**C**,**D**–**D1**,**F**–**F1**,**J1**,**K**–**L**) correspond to digital magnification showing IIIG9 detected in the basal bodies, and between the ring of nine outer microtubule duplets and the membrane of a motile cilia (arrow) (complementary images and results from [6,68]).

In rats, IIIG9 expression is found in ciliated tissues, such as the trachea, testicle, and brain [1,6]. In humans, IIIG9 was found to be within the top 200 upregulated genes in ependymal cells [58], and its localization has been associated with the cilia axoneme [59]. In addition, the analysis of IIIG9 in the BioGPS database shows high levels of mRNA in different areas of the CNS, such as the substantia nigra (pars compacta) [60,61], the anterodorsal and anteroventral nuclei of the thalamus, Barrington’s nucleus, and the bed nucleus of the stria terminalis [62,63,64]. In situ hybridization studies in rat and mouse brains have shown that IIIG9 mRNA is concentrated in the ependymal cells of the lateral ventricles, the dorsal third ventricle, and the dorsal fourth ventricle (Allen Brain Atlas [65]), as well as in the neurons of the hippocampus and Purkinje cells of the cerebellum to a lesser extent [1] (Figure 4A–C,D,D1,F,F1). We have reported the ciliary localization of IIIG9 in ependymal cells of the different cerebral ventricles (Figure 4B1), observing a dotted and discontinuous localization positioned on an edge of the cilium through super-resolution SIM (Figure 4B2). At the ultrastructural level, IIIG9 is located in the basal bodies and between the ciliary membrane and the axoneme of peripheral doublets of the ciliary microtubular structure 9 + 2 [6] (Figure 4H,H1,J–L). This dotted and discontinuous pattern is also preserved in primary cultures of ependymal cells disaggregated to the state of a single cell whose multiple cilia beat in a coordinated manner [6]. The ciliary localization of IIIG9 is similar to that of PP1 reported in the Chlamydomonas flagellum, where PP1 is a structural and functional part of the ciliary axoneme that is mainly localized in the central pair and (to a lesser extent) on the outer pairs near substrates such as IC138 and dynein I [66]. Hence, IIIG9 could play a significant role in guiding PP1 towards substrates that drive ciliary motility. In this way, the identification of new ciliary proteins, such as IIIG9, present in motile cilia may suggest important functions during human development, female and male fertility, airway protection and function, and cerebrospinal fluid circulation [67]. Furthermore, the absence of IIIG9 may lead to ciliopathies, such as hydrocephalus.

Many, or perhaps all, of the ciliary proteins are also located beyond the cilium with extraciliary functions that include the regulation of the cell cycle, cytoskeleton, and intracellular protein trafficking. In a genome-wide functional analysis of 24,373 independently depleted genes using siRNAs in the U2OS osteosarcoma cell line, IIIG9 was identified as a potential regulator of the cell cycle; since the downregulation of IIIG9 resulted in G_2_/M cells with large nuclei [69]. However, the underlying mechanism is unknown.

We recently proposed a new function for IIIG9 related to the maintenance of adherens junctions in the ependymal epithelium [68] (Figure 4I). Using immunogold electron microscopy analysis, we demonstrated that IIIG9 is localized near adherens junctions, similarly to that reported for PP1 alpha in proteomic analyzes of the E-cadherin interactome in human gastric carcinoma cells [70]. The in vivo loss of function in the adult ependymal epithelium of the lateral ventricle (produced by the stereotaxic injection of adenoviruses that express an interfering RNA for IIIG9) generates a partial ependymal denudation where areas of the ventricular wall are observed with the absence of cells, the direct exposure of the subependymal parenchyma to the CSF, and an evident ventriculomegaly. However, future research is expected to define whether the loss of function of IIIG9 in ependymal cells is associated with the loss of function of PP1 at cell junctions and whether ependymal denudation occurs due to changes in the phosphorylation/dephosphorylation levels of adherens junctions proteins. In the remaining ependymal cells, two different cell populations can be observed: (i) cells with a balloon-like morphology, the cytoplasmic localization of cadherins, and the expression of the cleaved caspase-3 cell death marker; (ii) polarized ependymal cells with “ciliary rigidity” [68]. Similar effects have been reported in loss-of-function studies targeting proteins that are part of the central and peripheral axoneme of 9 + 2 motile cilia, inhibiting cilia bending and leading to vibrational beating [71]. This condition in the cerebral ventricular system would prevent CSF clearance, contributing to its accumulation and ventriculomegaly as a primary event of hydrocephalus. Thus, IIIG9 expression in the differentiated ependyma, and potentially during their development from the radial glia, may be an essential phenomenon to limit the development of ventricular pathologies, such as congenital hydrocephalus.

We also first reported IIIG9 protein in the soma of pyramidal neurons of the hippocampus and in Purkinje cells from the cerebellum (Figure 4E,E1,G,G1). A positive correlation with the expression of mRNA was previously reported [1], suggesting that IIIG9 may play a hitherto unknown role in mature neurons.

## 7. Expression of IIIG9 during CNS Development

The early and high expression of IIIG9 in the ventricular and cortical wall at embryonic day 17 of the rat brain has been demonstrated by immunocytochemical studies in rat brains [6]. At this stage, IIIG9 is widely expressed throughout the ventricular and cortical thickness, and it is preferentially polarized at the apical border of the lateral ventricle, which houses the body of the neural stem cell, called the radial glia. Our localization results are also supported by data present in the CORTECON information base, which is a repository of cortical developmental gene expression, showing that the expression of IIIG9 mRNA (*PPP1R32*) is associated with neural differentiation states and the cortical specification of the upper cortical layers [72,73]. Moreover, evidence suggests that IIIG9 could participate in the development of neural stem cells (NSCs) by maintaining the signaling pathways for cell survival and differentiation, as demonstrated by DICER (riboendonuclease in the small RNA pathway) loss-of-function studies in NSCs, which increases anti-survival and/or apoptotic proteins and signaling pathways, thus leading to NSC cell death in the absence of mitogens [74]. Proteomic analysis showed that the levels of approximately 2900 proteins, including IIIG9, are affected. Therefore, the proteins lost in DICER-null NSCs could be relevant to or a consequence of the deregulation of cell survival and differentiation signaling pathways in NSCs or radial glia. Similarly, the location of apically polarized IIIG9 in the radial glia of the embryonic ventricular wall may be indicative of its role in cell polarization mechanisms that maintain adherens junctions or induce the multiciliogenesis of the radial glia that become ependymal cells. The general view is that multiciliated cells arise from progenitors after the inhibition of Notch, a process that triggers the activation of the master regulators of multiciliogenesis, GEMC1, and multicilin, which activate gene expression by binding the transcription factors, E2F4/5 and DP1 [75,76]. Additionally, the downstream binding of the transcription factor RFX2/3 mediates the biogenesis of both primary and motile cilia, while the transcription factor FOXJ1 anchors basal bodies, axoneme growth, and ciliary motility [77,78,79]. Notably, the analysis of the *IIIG9* gene locus in mice (chromosome 19) using the GTRD [80,81], IFTI [82], and PROMO [83] databases has identified the binding sites for the transcription factors RFX2/3 and FOXJ1 immediately and 408 bp upstream of the start codon, respectively. This in silico analysis complements expression and localization data, strengthening the idea that IIIG9 is involved in the multiciliated program. Furthermore, a ~14-fold increase in IIIG9 mRNA levels was detected just 5 h after the induction of multicilin in epithelial explants of *Xenopus laevis*, positioning IIIG9 as one of the top 15 genes upregulated among >500 mRNAs after treatment [75].

IIIG9 could also be part of the basal body of the primary cilium of the radial glia that contacts the ventricular cavity, given that the IIIG9–GFP fusion protein was shown to be localized at the base of the cilium from the mouse kidney collecting duct cell line, IMCD3 [84]. This suggests that IIIG9 is not an exclusive protein of motile cilia, as it can also be present in the primary cilium of different cells of an organism. Thus, like many ciliary proteins, IIIG9 has extraciliary functions, such as cell cycle regulation in tumor cells, the maintenance of adherens junctions in ependymal epithelium, and the genesis and differentiation of neuronal subpopulations during the development of the cerebral cortex.

## 8. Role of IIIG9 in Pathologies

Although there is little information that directly relates the deregulation of the *C11orf66* gene or the IIIG9 protein with the development of human pathologies, the following sections summarize what has been reported to date.

### 8.1. Ciliopathies

Ciliopathies are a set of developmental (degenerative) disorders of a single gene whose dysfunction alters the function of any type of cilia. According to the “Rare Diseases AutoRIF ARCHS^4^ Predictions” database [85,86], PPP1R32 dysfunction is postulated to be connected with the development of bronchiectasis-ciliary dyskinesia, cranioectodermal dysplasia, multifocal heterotopia, primary ciliary dyskinesia, situs inversus, and ciliary immobility associated with sterility. Similarly, the ciliary localization of IIIG9 has been demonstrated in human airway epithelial cells [4]. Furthermore, the *C11orf66* gene is one of the differentially expressed genes in the pseudoglandular-to-canalicular transition of human lung development [87], which could have broader implications in terms of lung function in both health and illness. Similarly, we have shown that the loss of IIIG9 in the adult ventricular wall of the nervous system induces the presence of non-polarized ependymal cells (balloon-like morphology) and ependymal cells with rigid cilia that probably vibrate, which may be the basis of the observed ventriculomegaly [68], and are key agents in the development of a ciliopathy, such as hydrocephalus.

### 8.2. Infertility

IIIG9 was first reported as a ciliary protein in the testes and oviducts [4,6]. In the testes, IIIG9 has been detected in mouse spermatogonia (GC-1), uniformly in the nucleus and cytoplasm; in mature bovine spermatozoa, IIIG9 is absent in the equatorial region of the head and midpiece, standing out in the tail and head and constituting a regulatory protein of interest in sperm development [28]. To date, the effects of IIIG9 inhibition on sperm development and capacitation are unknown. Both processes were studied using an ex vivo model of rat seminiferous tubules against exposure to two fungicides (carbendazim and iprodione) that produce a strong alteration of spermatogenesis that affects the migration of germ cells through the seminiferous tubules; it also impacts androgen synthesis and alters meiosis. In this study, increased IIIG9 expression was observed after 14 days of exposure to carbendazim (50 nM), decreasing after 21 days of exposure when combined with iprodione in equimolar proportions. In addition, IIIG9 was part of a series of proteins that were identified as potential markers of testicular dysfunction and infertility [88].

The targeted interruption of the *Ppp1cc* gene (PP1 gamma) also causes infertility in mice as a result of altered spermatogenesis coinciding with a loss of the transition between round and elongated spermatogonia and a decrease in germ cells, especially spermatids. These effects culminate in the generalized absence of sperm, resulting from apoptosis that occurs in all layers of the seminiferous tubules [89,90,91,92]. Thus, IIIG9 is likely a PP1 gamma regulatory protein that regulates its function to promote normal spermatogenesis.

### 8.3. Autism Spectrum Disorder (ASD)

ASD is a condition related to brain development that affects how the person perceives and socializes with other people, leading to problems in social interaction and communication, as well as the manifestation of restricted or repetitive behaviors, interests, and activities. Mak et al., 2017 carried out chromosomal microarray analysis in a cohort of 258 Chinese children with ASD, analyzing the copy number variants (CNVs) to identify possible molecular markers that predispose ASD development [93]. This study was focused on nine patients with pathogenic and probably pathogenic CNVs who manifested ASD at different ages. In one patient (number 9), who was diagnosed at 29 months of age without physical abnormalities and with normal cognitive function, there were two consecutive duplications in a mosaic pattern, 11q12.1-q12. 2 (5.55 Mb) and 11p11.2 (0.29 Mb), which extended through the centromere [93]. In the first duplication (5.55 Mb region), the *PPP1R32* (IIIG9) gene was one of the 64 genes present in this CNV that was classified as pathogenic and with unknown inheritance. Furthermore, this result was indicative of a marker chromosome, confirmed by karyotyping mos 47,XY,+mar/46,XY. Another patient with similar duplications (variant ID NSSV582454 present in the Children’s Hospital of Philadelphia database) presented a global developmental delay (GDD) and a delay in speech and language development [93]. These results could be related to the putative role of IIIG9 in the neural precursors of the cerebral cortex due to the high expression of this gene observed in the CORTECON database. In this way, questions arise regarding to the functional importance of IIIG9 during brain development and how the deregulation of this protein can contribute to the development of pathologies and developmental disorders, such as ASD.

### 8.4. Drug Use Disorders

Two reports have related IIIG9 expression to drug use disorders, such as alcoholism and cocaine use. Alcoholism is a complex disease consisting of the inability to control alcohol consumption due to physical and mental dependence. Although it has been established that genetic and environmental factors are crucial for its manifestation, the interaction between them is currently unknown. The screening analysis of the single-nucleotide polymorphisms (SNPs) of alcoholism using an Ensemble Bayesian Network (an important method used to analyze SNPs related to complex diseases) was used to identify the *IIIG9* gene (*C11orf66*) among 18 genes that are potentially correlated with vulnerability to alcoholism [94]. Interestingly, the authors of this study also identified compounds that could be used as potential medicines for the treatment of alcoholism since they regulate the expression of at least 14 of the 18 initially recognized genes. For *C11orf66*, 13 compounds were identified: four strongly increase gene expression, and five, including chloroprene, inhibit expression [94]. However, the carcinogenic and mutagenic effects of chloroprene limit its use in future molecular strategies to regulate the expression of IIIG9 in patients with alcoholism.

The mesolimbic system also plays an important role in neuroadaptation against drug use, mainly in the reward circuit that connects the ventral tegmental area (VTA) and the nucleus accumbens (NAc) [95]. In an RNA-seq transcriptomic analysis study, changes in VTA and NAc expression were analyzed in Rhesus macaques exposed to cocaine self-administration (3 months at a maximum of 3 mg/kg weight) and compared to a control group. IIIG9 (*PPP1R32*) was differentially expressed (*q* < 0.05) in the VTA of animals that consumed cocaine [96]. This increase in the expression of some genes is attributed to epigenetic changes and chromatin remodeling at the level of the dopaminergic system, and it is related to the transition from drug use to abuse [97]. In this way, IIIG9 is considered a gene of interest in understanding the molecular and cellular mechanisms of addiction. Future studies will help to understand whether regulating IIIG9 function can become a potential treatment strategy.

### 8.5. Tumors

The Human Protein Atlas database [57] reports the expression of IIIG9 mRNA in cancerous human tissues, including endometrial, ovarian, breast, and lung cancers. Additionally, IIIG9 was found to be one of the top 10 upregulated genes in the human pancreas ductal carcinoma cell line, PANC-1, in response to metformin [98], indicating that its expression is subject to regulation, although the effect is still unknown.

In brain tumors, the clearest evidence is related to the development of ependymomas, which are the third most frequent type of brain tumor. In recent years, researchers have worked hard to classify the different subtypes of existing ependymomas through transcriptomic and genomic studies and the analysis of gene methylation profiles that have been shown to be related to the region in the CNS where these tumors grow [99]. At present, there are at least 10 different groups of ependymomas that are grouped into four subtypes that develop in the walls of the central canal of the spinal cord. Three subtypes grow in the posterior fossa of the fourth ventricle and three other subtypes, also known as supratentorial (ST) ependymomas, develop in the walls of the lateral ventricles [100,101,102,103]. Although the expression and function of IIIG9 in the different classes of ependymomas are unknown at present, the analysis of the information available on these subtypes of tumors can shed some light on this question. In this context, a subtype of human ST anaplastic ependymomas (grade 3) called *RELA* fusion ependymomas (the fusion is called *ZFTA-RELA*, comprising the gene *c11orf95*, recently designed as *ZFTA*, and the gene encoding the main effector of canonical NF-κB signaling), representing about 60–70% of tumors that grow at the level of the lateral ventricles, were shown to present a chomothripsis event in a region of chromosome 11 (segments 11q12.1-11q13.3) where the IIIG9 gene (locus 11q12.2 for *C11orf66*) is found. ST ependymomas predominantly occur in young children and adolescents, and they have a poor clinical prognosis since they are highly aggressive and metastatic. Recently, the role of the epithelial–mesenchymal transition (EMT) has become seriously considered in CNS tumors. Specifically, in RELA fusion-type ST ependymomas, there is an upregulation of *N*-cadherin mRNA levels, along with SNAI1/Snail, SNAI2/Slug, and ZEB1, which are genes related to the EMT and downregulation of E-cadherin [104]. Similarly, a comparative transcriptomic analysis of grade 2 ependymomas (intermediate risk) and grade 3 ependymomas (high risk) showed that in the latter, there is a decrease in the regulation of CDH-1 (E-cadherin) and the activation of this machinery of destruction. This suggests a weakening of intercellular junctions and the dissociation of the cytoskeleton, facilitating tumor growth and progression [104]. This last characteristic is closely related to the evidence recently reported by our group showing that IIIG9 is a protein located at the adherens junctions of adult ependymal cells and that the loss of its function promotes the presence of nonpolarized cells in the ventricular walls of the lateral ventricle, with a low and delocalized expression of cadherins and an increase in the expression of the cell death marker, active caspase-3 [68]. Thus, these data lead us to speculate that, even though the mechanism of cell death may be the result of the absence of IIIG9 expression in the differentiated adult ependyma, under the tumorigenic context of the development of ST ependymomas, IIIG9 loss of function may be an event that promotes the lack of cell adhesion and the dissemination of these tumor cells frequently observed in this type of tumor.

**Figure 5:**
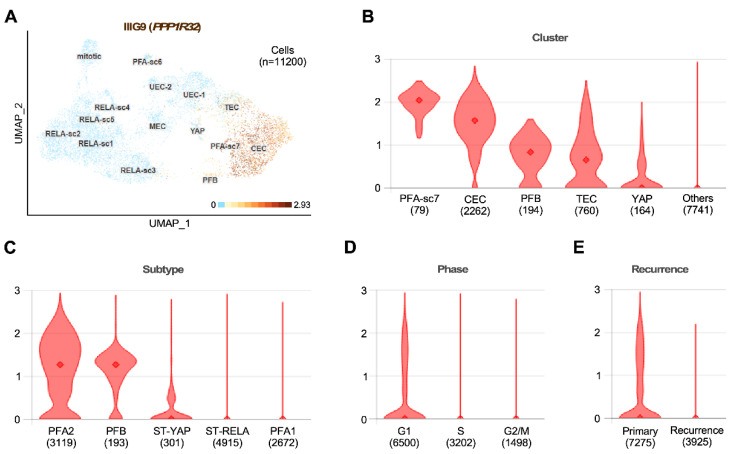
IIIG9 expression is restricted to ciliated ependymoma cells of the posterior fossa. (**A**) Harmony alignment projection of scRNA-seq analysis of 26 pediatric patients with ependymoma (EPN). IIIG9 (*PPP1R32*) expression is shown in the brown color range and corresponds to the values generated by the adaptive threshold low-rank approximation (ALRA). (**B**–**E**) IIIG9 expression ALRA range is shown by cluster (**B**), subtype (**C**), phase (**D**), and recurrence (**E**) The number of cells in each selection is placed in brackets (*n* = 11200 cells). Seven subgroup clusters of posterior fossa group A (from PFA-sc1 to PFA-sc7), five subgroup clusters of *ZFTA-RELA* group (from RELA-sc1 to RELA-sc5), one posterior fossa group B (PFB), and one YAP-MAMLD1 group (YAP) are shown. Five PFA subgroup clusters correspond to ciliated EPN cells (CEC), transporter EPN cells (TEC), mesenchymal EPN cells (MEC), and undifferentiated EPN cells 1 and 2 (UEC-1 and EC-2, respectively); cells are in mitosis (mitotic). Uniform manifold approximation and projection (UMAP) was used for classification. The database was generated by Gillen and colleagues [105] and is available in the browsable web resource of the full EPN scRNA-seq dataset at the Pediatric Neuro-oncology Cell Atlas [107].

Unlike ST ependymomas, tumors that develop at the level of the posterior fossa are often found in adolescent children and older people. Two clinical subgroups have been described: PFA (posterior fossa A ependymomas) and PFB (posterior fossa B ependymomas); compared with the preference for expression of ciliary genes observed in PFB, PFA has a worse clinical prognosis than PFB and presents a genetic program associated with the expression of proteins found in inflammation signaling pathways. In the recently generated database described by Gillen et al., 2020, who analyzed single-cell RNA expression from 26 childhood ependymoma samples, PPP1R32 expression in PFA ependymomas was associated with a higher differentiation status, such as that observed in ciliated EPN cells (CEC) (Figure 5) [105]. In contrast, PPP1R32 expression is absent in almost all cells with a low cell differentiation status, such as undifferentiated cells (UEC1 and 2), mesenchymal cells (MEC), and highly mitotic cells [105]. These results are supported by the expression evidence presented above, which indicates that IIIG9 is highly expressed in the human cell line AF22, which corresponds to NSCs generated from iPSCs [106]. In addition, in samples from different RELA ependymomas, the expression of PPP1R32 was found to be significantly low (close to 1%), which is in line with our hypothesis that in this type of ependymoma, the loss of IIIG9 expression and function may be a key factor for its metastatic phenotype.

## 9. Conclusions and Perspectives

Protein phosphorylation and dephosphorylation reactions are dynamic regulatory mechanisms that change in time and space and that support the development of diverse biological functions that are fundamental for cellular activities. Thus, protein kinases (about 500 proteins in humans) and phosphatases (about 140 protein phosphatases in humans) orchestrate and regulate the function of hundreds to thousands of proteins at different times. PP1 is a phosphatase that is widely used in cell economy, where it is estimated that it is capable of catalyzing between 30% and 50% of the total dephosphorylation reactions that occur in cells, regulating various physiological and pathological processes. Given the diversity of functions and processes attributed to PP1, its specificity and regulation are determined by the formation of protein complexes between the catalytic subunits of PP1 (α, β, and γ), with highly specific regulatory subunits that determine where and when PP1 acts. IIIG9 is the regulatory subunit 32 of PP1, whose expression was initially restricted to highly polarized, ciliated epithelia, such as those of the trachea, oviducts, testes, and ventricular brain walls. We now know that IIIG9, like other ciliary proteins, may have extraciliary functions, such as the maintenance of adherens junctions in the epithelium, where it is expressed as ependymal cells. We also know that the loss of its function triggers the loss of these junctions, the apoptosis of ependymal cells, and the beginning of dilation of the cerebral ventricular system due to a possible deterioration in cerebrospinal fluid circulation (ventriculomegaly), which is an initial stage of development of hydrocephalus. In turn, the literature suggests that IIIG9 is a versatile protein that may participate in cellular processes, such as the cell cycle, the assembly and/or function of primary (immotile) cilia and motile cilia, neural differentiation states, and the cortical specification of the upper cortical layers, where the action of PP1 could be necessary. Finally, IIIG9 may be a key protein in promoting the development of pathologies, such as various ciliopathies (e.g., infertility and hydrocephalus), developmental disorders of the nervous system (e.g., ASD), drug abuse and dependence (e.g., alcoholism and cocaine addiction), and the development of brain tumors with a poor clinical prognosis, due to their ability to spread within and outside the CNS (e.g., anaplastic supratentorial ependymomas and type A posterior fossa ependymomas). The protumoral effect of loss of IIIG9 expression could be explained by the loss of cell–cell junctions, favoring the metastasis of tumor cells. However, these and many other questions regarding the role of IIIG9 in human physiology and how its deregulation contributes to the development of different diseases will continue to be the subject of further research.

## Figures and Tables

**Table 1:** IIIG9-interacting proteins.

Proteins	Experimental Approach	Ref.
AKAP8L, ALS2CL, ATN1, ATPAF2, B9D2, BAG4, BCAS2, C10orf55, CATSPER1, CCDC57, COG6, COX5B, CSTF2, CTDSP1, CYSRT1, DTX2, FAM168B, FHL2, FHL3, FHL5, FKBP6, FRS3, GOLGA2, HGS, HNRNPH1, HOMER3, HOXA1, HSF2BP, INCA1, KCTD9, KPRP, KRT31, KRTAP13-2, KRTAP19-5, KRTAP19-6, KRTAP3-1, KRTAP3-2, KRTAP6-2, KRTAP6-3, KRTAP8-1, LASP1, LMO2, LMO4, METTL27, MYO15B, OIP5, OTUD7B, PFDN5, PLA2G10, PLEKHG4, PLEKHN1, PPP1CC, PRDM14, PRKAA2, QARS1, RBM11, RFX6, RIMBP3C, SLAIN1, TEKT4, TFG, TGM7, TRAF1, TRAF2, TRIB3, TSC1, UNKL, VMAC, WBSCR27, WWOX, and ZMYND12	Two-hybrid	[30]
APP	Reconstituted complex	[36]
DVL2	Affinity Capture—MS	[37]
GAPDHS, GMPPB, GPX1, IDE, ISCA2, KLHL123, KLHL22, KLHL8, KLHL9, MPP7, SAMD10, UBR1, UBR2, UBR3, USP7, ZER1, and ZYG11B	Affinity Capture—MS	[38,39]
PPP1CA	Affinity Capture—Western	[16]
PPP1CA and PP1CC	Two-hybrid; reconstituted complex	[27]
RNF123	Affinity Capture—MS	[40]
RPL21	Proximity Label—MS	[41]

Source: BioGRID^4.4^, The Database of Protein, Genetic, and Chemical Interaction [34,35].

## Data Availability

The data that support the findings of this study are available from the first author (L.H.) upon reasonable request.

## Supplementary Materials

The following supporting information can be downloaded at: https://www.mdpi.com/article/10.3390/cells11203327/s1. Table S1. Related Pathways or Biological Functions with IIIG9 interactors.
