# Supplementary material for: Is IIIG9 a New Protein with Exclusive Ciliary Function? Analysis of Its Potential Role in Cancer and Other Pathologies
Source: Cells. 2022 Oct 21;11(20):3327. doi: 10.3390/cells11203327 (PMC9600092; doi:10.3390/cells11203327)
Supplement: Supplementary file 1 [file cells-11-03327-s001.zip › cells-1899835-supplementary.pdf]

Table S1. Related Pathways or Biological Functions with IIIG9 interactors.

IIIG9 Interactors	Related Pathways or Biological Functions.	Reference
CYSRT1	Identical protein binding	[30]
KRT31	Keratinization and Development Biology.	[43-45]
BAG4	TWEAK pathway and death receptor signaling.	[43-45]
TEKT4	Sperm Motility	[43-45]
HSF2BP	Meiosis. Spermatogenesis and fertility.	[43-45]
TRAF1	TWEAK pathway and apoptotic pathway triggered by HIV1.	[43-45]
FKBP6	Cell Cycle, Mitotic and Meiosis.	[43-45]
GOLGA2	Neurodegenerative diseases and PLK1 signaling events.	[43-45]
TRAF2	IL-4 signaling pathways and TWEAK pathway.	[43-45]
KRTAP6-2	Keratinization and development Biology.	[43-45]
FHL3	Actin Binding. Transcriptional coactivator of FOXK1.	[43-45]
KRTAP6-3	Keratinization and development Biology.	[43-45]
ATPAF2	-	[43-45]
RFX6	Regulation of beta-cell development and development biology.	[43-45]
HOXA1	Activated PKN1 stimulates transcription of AR (androgen receptor) regulated genes KLK2 and KLK3. Mesodermal Commitment Pathway.	[43-45]
HGS	Tyrosine kinases adaptors and Budding and maduration of HIV maduration.	[43-45]
COG6	Vesicle- mediated transport and Transport to the Golgi and subsequent modification.	[43-45]
VMAC	-	[43-45]
HOMER3	Protein-Protein Interactions at synapses and Transmission across Chemical Synapses.	[43-45]
PLEKHG4	Possible role in intracellular signaling and cytoskeleton dynamics at the golgi.	[43-45]
ATN1	Transcriptional corepressor. Protein domain specific binding and toxin activity.	[43-45]
LMO2	Embryonic and Induced Pluripotent Stem Cells and Lineage- specific Markers. Hematopoietic Stem Cell Differentiation.	[43-45]
WWOX	Signaling by ERBB4 and gene expression.	[43-45]
KRTAP3-1	Keratinization and development Biology	[43-45]
PRKAA2	Metabolism and RET signaling.	[43-45]
FAM168B	Inhibitor of neuronal axonal outgrowth.	[43-45]
KRTAP13-2	Keratinization and development Biology	[43-45]
RIMBP3C	Sperm development.	[43-45]
PFDN5	Chaperonin-mediated protein folding and Metabolism of proteins.	[43-45]
DTX2	Constitutive Signaling by NOTCH and Signaling GPCR.	[43-45]
TFG	Vesicle- mediated transport and Transport to the Golgi and subsequent modification.	[43-45]
AKAP8L	RNA binding and DEAD/H-box RNA helicase binding.	[46]
LMO4	Embryonic and Induced Pluripotent Stem Cells and Lineage- specific Markers and Neuroscience.	[43-45]
KRTAP19-5	Keratinization	[43-45]
LASP1	Cytoskeletal signaling and Aquaporin-mediated transport.	[43-45]
CCDC57	Centriole duplication, mitosis and ciliogenesis.	[43-45]
FRS3	NGF Pathway and Cytokine Signaling in Immune system.	[43-45]
CSTF2	Processing of Capped Intrinless Pre-RNA and tRNA processing.	[43-45]
PRDM14	Mesodermal Commitment Pathway and Transcriptional regulation of pluripotent stem cells.	[43-45]
KPRP	Keratinization differentiation.	[43-45]
COX5B	Metabolism and TP53 regulates metabolic genes.	[43-45]
TSC1	mTOR signaling pathway and RET signaling.	[43-45]
C10orf55	-	-
OIP5	Cromosome maintenance and Cell Cycle, Mitotic.	[43-45]
INCA1	Cyclin binding.	[43-45]
UNKL	Class 1 MHC mediated antigen processing and presentation and Innate Immune system.	[43-45]
PLA2G10	Metabolism and Acyl chain remodeling of PE.	[43-45]
KCTD9	Activation of cAMP-dependent PKA and Hepatic ABC transporters.	[43-45]
FHL2	Metabolism and Signaling events mediated by HDAC Class III.	[43-45]
BCAS2	mRNA splicing-Major Pathway and Gene expression.	[43-45]
CTDSP1	mir-124 predicted interactions with cell cycle and differentiation and Coregulation of Androgen receptor activity.	[43-45]
METTL27	Methyltransferase activity.	[47]

KRTAP3-2	Keratinization.	[43-45]
KRTAP8-1	Keratinization.	[43-45]
KRTAP19-6	Keratinization.	[43-45]
HNRNPH1	MECP2 and associated Rett Syndrome and Signaling by FGFR2.	[43-45]
QARS	Metabolism and Peptide chain eleongation.	[43-45]
OTUD7B	TWEAK pathway and Ovarian tumor domain proteases.	[43-45]
PLEKHN1	Cardiopilin binding.	[48]
ALS2CL	Vesicle- mediated transport and RAB GEFs exchange GTP for GDP on RABs.	[43-45]
TGM7	Protein-glutamine gamma-glutamyltransferase activity.	[43-45]
CATSPER1	Fertilization ans Sweet Taste signaling.	[43-45]
SLAIN1	Cytoplasmic microtubule organization.	[47]
B9D2	Mitotic Prometaphase and Mitotic Metaphase and Anaphase. Gamma tubulin binding.	[43-45]
PPP1CC	Metabolism and Beta-Adrenergic Signaling. RNA binding and hydrolase activity.	[43-45]
ZMYND12	Metal ion binding activity.	[43-45]
RBM11	poly (U) RNA binding and cell differentiation.	[43-45]
TRIB3	Metabolism and RET signaling.	[43-45]
MYO15B	ATP binding activity, actin binding activity and cytoskeletal motor activity.	[43-45]
FHL5	Transcription CREM signaling in testis.	[43-45]
APP	Peptide ligand- binding receptors. Neurodegenerative Diseases.	[43-45]
DVL2	Wnt mediated activation of DVL. DNA damage response.	[43-45]
IDE	A- beta plaque formation and APP metabolism. Metabolism of proteins.	[43-45]
PPP1CA	Metabolism and beta- adrenergic signaling.	[43-45]
RNF123	Class 1 MHC mediated antigen processing and presentation and Innate Immune system.	[43-45]
RPL21	Peptide chain elongation and metabolism.	[43-45]
UBR1	Class 1 MHC mediated antigen processing and presentation and protein ubiquitylation.	[43-45]

References

30. Luck, K.; Kim, D.K.; Lambourne, L.; Spirohn, K.; Begg, B.E.; Bian, W.; Brignall, R.; Cafarelli, T.; Campos-Laborie, F.J.; Charloteaux, B.; et al. A reference map of the human binary protein interactome. *Nature* **2020**, *580*, 402-408, doi:10.1038/s41586-020-2188-x.

43. Belinky, F.; Nativ, N.; Stelzer, G.; Zimmerman, S.; Iny Stein, T.; Safran, M.; Lancet, D. PathCards: multi-source consolidation of human biological pathways. *Database (Oxford)* **2015**, *2015*, 1-13, doi:10.1093/database/bav006.

44. Safran, M.; Rosen, N.; Twik, M.; BarShir, R.; Stein, T.I.; Dahary, D.; Fishilevich, S.; Lancet, D. The GeneCards Suite. In *Practical Guide to Life Science Databases*, Abugessaisa, I., Kasukawa, T., Eds.; Springer Singapore: Singapore, 2021; pp. 27-56.

45. PathCards, Pathway Unification Database. Available online: https://pathcards.genecards.org/ (accessed on 10/6/2022).

46. Westberg, C.; Yang, J.P.; Tang, H.; Reddy, T.R.; Wong-Staal, F. A novel shuttle protein binds to RNA helicase A and activates the retroviral constitutive transport element. *J Biol Chem* **2000**, *275*, 21396-21401, doi:10.1074/jbc.M909887199.

47. Gaudet, P.; Livstone, M.S.; Lewis, S.E.; Thomas, P.D. Phylogenetic-based propagation of functional annotations within the Gene Ontology consortium. *Brief Bioinform* **2011**, *12*, 449-462, doi:10.1093/bib/bbr042.

48. Sano, E.; Shono, S.; Tashiro, K.; Konishi, H.; Yamauchi, E.; Taniguchi, H. Novel tyrosine phosphorylated and cardiolipin-binding protein CLPABP functions as mitochondrial RNA granule. *Biochim Biophys Acta* **2008**, *1783*, 1036-1047, doi:10.1016/j.bbamcr.2007.12.009.
